# 

**Published:** 1890-07

**Authors:** 


### HALL'S JOURNAL OF HEALTH

ESTABLISHED 1854

CONTENTS.

VOL. 37. No. 7.

| | PAGE. |
|---|---|
| Special | 145 |
| Alcoholic Adulteration | 145 |
| The Eyes | 147 |
| Government Getting us Good Groceries | 150 |
| Apples as Medicine | 152 |
| A Presentiment | 153 |
| The Invisible World | 154 |
| What Man is Made of | 155 |
| True Worth Recognized | 155 |
| Another Boy Prodigy | 156 |
| Great Times for the Widows | 156 |
| Mind Reading | 157 |
| A Pathetic Scene | 158 |
| Evil Effects of Catarrh | 159 |
| The Nose | 159 |
| Molly Fancher's Bazaar | 161 |
| Poison for Arrow Tips | 161 |
| Private Benevolent Societies and the Census | 162 |
| Miscellaneous | 163 |
| A Gratifying Acknowledgment | 163 |
| Candies made of White Clay | 163 |
| Cure for Drunkenness | 164 |
| The Potato | 164 |
| Paste for Scrap Books | 165 |
| Literary | 167 |

INDEX TO ADVERTISEMENTS OF HALF A PAGE AND OVER.

| | PAGE. |
|---|---|
| Woman's Work | I |
| Celluloid | II |
| Hollow Suppositories | III |
| Hospital Remedy Co | IV |
| Hall's Journal of Health | V |
| Goldsmith's Pianos | V |
| E. C. Morris & Co | VI |
| Dennin's Certain Cure | VII |
| The Victor Safe and Lock Co | VII |
| Revised Premium List | VIII |
| Herba-Vita Remedy Co | IX, X |
| Christ Before Pilate | XI |

